# Consequences of Lethal-Whole-Body Gamma Radiation and Possible Ameliorative Role of Melatonin

**DOI:** 10.1155/2014/621570

**Published:** 2014-11-05

**Authors:** Ehsan Mihandoost, Alireza Shirazi, Seied Rabie Mahdavi, Akbar Aliasgharzadeh

**Affiliations:** ^1^Department of Medical Radiation Engineering, Science and Research Branch, Islamic Azad University, Tehran, Iran; ^2^Department of Medical Physics and Biomedical Engineering, Faculty of Medicine, Tehran University of Medical Sciences, Tehran, Iran; ^3^Department of Medical Physics, Faculty of Medicine, Iran University of Medical Sciences, Tehran, Iran; ^4^Department of Radiology and Medical Physics, Faculty of Paramedicine, Kashan University of Medical Sciences, Kashan, Iran

## Abstract

Gamma radiation induces the generation of free radicals, leading to serious cellular damages in biological systems. Radioprotectors act as prophylactic agents that are administered to shield normal cells and tissues from the deleterious effects of radiation. Melatonin synergistically acts as an immune-stimulator and antioxidant. We investigated the possible radioprotective role of melatonin (100 mg/kg i.p.) against lethal-whole-body radiation- (10 Gy) induced sickness, body weight loss, and mortality in rats. Results of the present study suggest that exposure to lethal-whole-body radiation incurred mortality, body weight loss, and apoptosis and it also depleted the immunity and the antioxidant status of the rats. Our results show that melatonin pretreatment provides protection against radiation induced mortality, oxidative stress, and immune-suppression. The melatonin pretreated irradiated rats showed less change in body weight as compared to radiation only group. On the other hand, melatonin appeared to have another radioprotective role, suggesting that melatonin may reduce apoptosis through a caspase-3-mediated pathway by blocking caspase-3 activity.

## 1. Introduction

Ionizing radiation is commonly used in diagnostic, therapeutic, and industrial settings. However, the damaging effects of radiation restrict its applications. Therefore, the concept of radiation consequences is important in medicine and other related occupations [1].

Today, researches for new strategies to prevent radiation damage are in progress. Some of these efforts are based on prevention from oxidative stress, as it is the main factor responsible for radiation-induced damage [2]. Ionizing radiation interacts with biological systems to produce free radicals or reactive oxygen species (ROS), which attack various cellular components including DNA, proteins, and membrane lipids, leading to serious cellular damage [3]. To control the onset of ROS, cells have developed their own antioxidant defense system, which includes enzymatic and nonenzymatic components. The antioxidant system consists of low-molecular-weight antioxidant molecules, such as glutathione (GSH), melatonin, vitamin E, uric acid, and various antioxidant enzymes [4]. For example, the antioxidant enzymes, superoxide dismutase (SOD), the first line of defense against oxygen-derived free radicals, catalyse the dismutation of the superoxide anion (O_2_^•−^) into hydrogen peroxide (H_2_O_2_). H_2_O_2_ can be transformed into H_2_O by glutathione peroxidase (GPx) and catalase (CAT) [5, 6]. It has been reported that nitric oxide (NO), as well as its derivatives, may play a role in multistage carcinogenesis [7]. NO reacts rapidly with the superoxide anion (O_2_^•−^) to form peroxynitrite (ONOO^−^), which in itself is cytotoxic and readily decomposes into the highly reactive and toxic hydroxyl radical (^•^OH) and nitrogen dioxide (NO_2_). ONOO^−^ is much more toxic than NO and O_2_^•−^ which causes diverse chemical reactions in biological systems including lipid peroxidation (LPO). One of the indices of oxidative damage is the malondialdehyde (MDA) formation as an end-product of LPO [7]. Lipid radicals are believed to be formed by the reaction of hydroxyl (^•^OH) radicals generated by ionizing radiation with polyunsaturated fatty acids, which can subsequently react with oxygen to form lipid peroxyl radical (LOO^•^), which can damage the DNA and cell compartments [3]. Thus, scavenging free radicals and inhibiting lipid peroxidation may be an important key to protection against oxidative stress caused by ionizing radiation.

Apoptosis is a programmed cell death (PCD) involving morphological changes such as DNA fragmentation. PCD is executed by a set of dormant cysteine-proteases, the caspases [8]. Caspase activation is a common response to ionizing radiation that actively initiates DNA fragmentation. Caspase-3, on activation, initiates fragmentation by translocating from the cytoplasm to the nucleus, where it cleaves genomic DNA at internucleosomal regions, generating oligonucleosomal fragments [2].

With the aim of protection against the damaging effects of radiation, various radioprotectors have been studied in several experiments and shown to have partial or complete success in protection [9]. Melatonin has been shown to be an immune-stimulator [10, 11] and antioxidant [12–14]. Melatonin synergistically acts as a direct free radical scavenger [15] and indirect antioxidant via its stimulatory actions on antioxidant enzymes activity and inhibitory actions on prooxidative enzymes activity [16]. Furthermore, due to small size and high lipophilicity, melatonin crosses membrane and reaches to all biological compartments of the cell [17].

Koc et al. [18] reported that pretreatment with melatonin prevented gamma radiation-induced damage on rat peripheral blood cells in vivo. Vijayalaxmi et al. [19] and Reiter et al. [20, 21] showed that melatonin reduces gamma radiation-induced chromosome damage, micronuclei and primary DNA damage in human peripheral blood lymphocytes in their in vitro and in vivo/in vitro studies. Moreover, radioprotective effect of melatonin against oxidative damages caused by irradiation has been reported in several organs [22, 23].

In the present study, we investigated the possible protective effects of pretreatment with melatonin (100 mg/kg i.p.) against lethal-whole-body radiation induced mortality, oxidative stress, and immune-suppression in rats. Moreover, the role of melatonin in reducing apoptosis through caspase-3-mediated pathway has been evaluated.

## 2. Materials and Methods

### 2.1. Chemicals

In this experimental study, all reagents were of the highest quality available. Melatonin was obtained from sigma Chemical Co. (St. Louis, MO, USA) and other chemicals used in this study were obtained from BioVision (980 Linda Vista Avenue, Mountain View, CA 94043, USA).

### 2.2. Animals

Adult male albino Wistar rats weighing 200–250 g were obtained from Experimental Animal Laboratory section of Department of Pharmacology, Tehran University of Medical Sciences, and were housed in stainless steel cages and supplied with wood chips, in a temperature controlled room (22°C) and a 12 h light-dark cycle. The animals were allowed a free access to tap water and standard diet for the duration of the study. The experimental protocol was in accordance with the guidelines for care and use of laboratory animals as adopted by the Ethics Committee of the School of Medicine, Tehran University of Medical Sciences, Tehran, Iran.

### 2.3. Experimental Design

Ninety rats were divided into six groups. Group 1 did not receive melatonin or irradiation and served as Control group (Con group). Group 2 did not receive melatonin or irradiation but received 500 *μ*L isotonic NaCl solution, intraperitoneally (i.p.) and served as vehicle group (Veh group). Group 3 only received 100 mg/kg melatonin (i.p.) before radiation time but did not receive any radiation and served as Melatonin only group (Mel group). Group 4 were only exposed to 10 Gy whole-body gamma irradiation and served as Radiation only group (Rad group). Group 5 received 500 *μ*L isotonic NaCl solution (i.p.) and were exposed to 10 Gy whole-body gamma irradiation and served as vehicle plus radiation group (Veh + Rad group). Group 6 received 100 mg/kg melatonin (i.p.) 30 minutes prior to radiation time and were exposed to lethal-whole-body irradiation of 10 Gy (Mel + Rad group).

Rats in groups 3 and 6 were given an intraperitoneal (i.p.) injection of freshly prepared melatonin in 500 *μ*L of 10% absolute ethanol solution in the evening. Melatonin was first dissolved in a small amount of absolute ethanol (50 *μ*L) and then diluted with isotonic NaCl solution in final ethanol concentration 10%. Also, the rats in groups 2 and 5 received 10% absolute ethanol in isotonic NaCl.

The concentration and selection of 30-minute interval between melatonin injection and exposure to gamma radiation were largely based on previous studies [14, 18, 24–28].

After 24 hours, five rats from each group were sacrificed under ether anesthesia and blood was collected from heart puncture. Each blood sample was divided into two parts. One part was used for lymphocyte count and caspase-3 evaluation and another part was used for measurement of the levels of MDA, NO, and TAC (total antioxidant capacity) and the antioxidant enzymes activities in serum. Serums were frozen at −20°C for the following measurements.

### 2.4. Survival and Body Weight Assay

Ten remaining rats from each group were kept for survival and body weight studies. The mortality of rats was monitored every other day for a month.

Body weights of the rats in all groups were observed weekly. The percent change in body weight in each group of rats was recorded every week by dividing the average body weight of those rats on the first day of experiment.

### 2.5. Irradiation

All of the rats (nonirradiated and irradiated groups) were anesthetized with an intraperitoneal injection of ketamine (50 mg/kg) and chlorpromazine (10 mg/kg), and then the rats in groups 4 to 6 were exposed to a lethal-whole-body gamma radiation dose of 10 Gy. Irradiation was performed using a cobalt-60 teletherapy unit (Theratron 780, Atomic energy of Canada limited, Canada) at a dose rate of 50 cGy/minute with SSD (source surface distance) method [SSD: 80 cm, field size (at SSD = 80 cm): 10 cm × 10 cm].

### 2.6. Biochemical Analysis

All of the parameters assessments were operated according to instructions of BioVision assay kits (980 Linda Vista Avenue, Mountain View, CA 94043 USA) and determined by a colorimetric method with ILISA Microplate Reader (Bio Tek Instruments, Inc., USA).

Nitric Oxide (NO) Colorimetric Assay Kit provides a measure of total nitrate or nitrite in two-step process. The first step converts nitrate to nitrite utilizing nitrate reductase. The second step uses Griess Reagents to convert nitrite to a deep purple azocompound. The amount of the azochromophore accurately reflects nitric oxide amount in samples. Nitric oxide level can be determined as a function of nitrate concentration by absorbance at 540 nm. The NO level was expressed as nitrate nmol/*μ*L.

Lipid Peroxidation Assay Kit provides a tool for sensitive detection of the MDA in a sample. The MDA in the sample is reacted with thiobarbituric acid (TBA) to generate the MDA-TBA adduct. The MDA-TBA adduct can be quantified colorimetrically at 532 nm and expressed as nmol/mL.

Superoxide Dismutase (SOD) Assay Kit, briefly, utilizes WST-1 that produces a water-soluble formazan dye upon reduction with superoxide anion. The rate of the reduction with a superoxide anion is linearly related to the xanthine oxidase (XO) activity and is inhibited by SOD. The activity of SOD can be determined by absorbance at 450 nm using a microplate reader. The SOD activity was also expressed as percent of inhibition rate (inhibition rate %).

Glutathione Peroxidase (GPx) Assay Kit, briefly, measures GPx activity through a coupled reaction with glutathione reductase (GR). In the assay, GPx reduces Cumene Hydroperoxide and oxidizes GSH to GSSG. The generated GSSG is reduced to GSH with consumption of NADPH by GR. The decrease of NADPH is proportional to GPx activity in the reactions. The decrease of NADPH can be measured by absorbance at 340 nm. The GPx activities were expressed as mU/mL (one unit is defined as the amount of enzyme that will cause the oxidation of 1.0 *μ*mol of NADPH to NADP^+^ under the assay kit condition per minute at 25°C).

Catalase (CAT), briefly, can be determined by this manner, catalase first reacts with H_2_O_2_ to produce water and oxygen, and the unconverted H_2_O_2_ reacts with OxiRed probe to produce a product, which can be measured at 570 nm. Catalase activity is reversely proportional to the signal. The CAT activity was also expressed as mU/mL (one unit of catalase is the amount of catalase decomposes 1.0 *μ*mol of H_2_O_2_ per min at pH 4.5 at 25°C).

Total Antioxidant Capacity (TAC) Assay Kit utilized Trolox to standardize antioxidants, with all other antioxidants being measured in Trolox equivalents. Measurement of the combined nonenzymatic antioxidant capacity of biological fluids and other samples provides an indication of the overall capability to counteract reactive oxygen species (ROS), resist oxidative damage, and combat oxidative stress. In this case Cu^++^ ion is converted to Cu^+^ by both small molecule and protein antioxidant. The reduced Cu^+^ ion is chelated with a colorimetric probe giving a broad absorbance peak around 570 nm, proportional to the total antioxidant capacity. TAC can be determined as a function of Trolox and expressed as Trolox nmol/*μ*L.

### 2.7. Lymphocyte Count (LC) and Caspase-3 Activity

Lymphocytes were isolated from each blood sample using Ficoll-Histopaque density gradients (Sigma, St. Louis, MO, USA) with modification. Blood was diluted 1 : 3 with phosphate buffered saline (PBS) and layered on to the Histopaque in the ratio of 2 : 1 (blood + PBS: Histopaque). The blood was centrifuged at 400 ×g for 20 min at room temperature. The lymphocytes layer was removed and then washed twice in PBS at 250 ×g for 10 min each. Liquid layer was removed and then 1 mL of PBS was added to sediment layer (lymphocytes layer) as a final sample. A thin layer of final sample was prepared on a glass slide and number of lymphocytes was counted by microscope (Olympus Optical Co. Ltd., Japan). LC was also expressed as 10^6^ cells/mL.

Lymphocytes collected in previous step were used for caspase-3 activity assay according to manufacturer's instructions. The Caspase-3 Colorimetric Assay Kit provides a means for assaying the activity of caspases that recognize the sequence DEVD. The assay is based on spectrophotometric detection of the chromophore* p*-nitroaniline (*p*NA) after cleavage from the labeled substrate DEVD-*p*NA. The* p*NA light emission can be quantified using a microtiter plate reader at 405 nm. The caspase-3 activity is presented as* p*NA optical density (OD)/10^6^ cells per mL.

### 2.8. Statistical Analysis

Each data point represents mean ± standard error on the mean (SEM) of at least five animals per group. A one-way analysis of variance (ANOVA) was performed to compare different groups, followed by Tukey's multiple comparison tests. *P* < 0.05 was considered to represent a statistically significant difference.

## 3. Results

Figures 1 and 2 show the results of survival and body weight studies. The NO and MDA levels, antioxidant enzymes activities and total antioxidant capacity (TAC) level, lymphocytes count (LC), and caspase-3 activity are given in Figures 3–10.

### 3.1. Survival Assay

**Figure 1:**
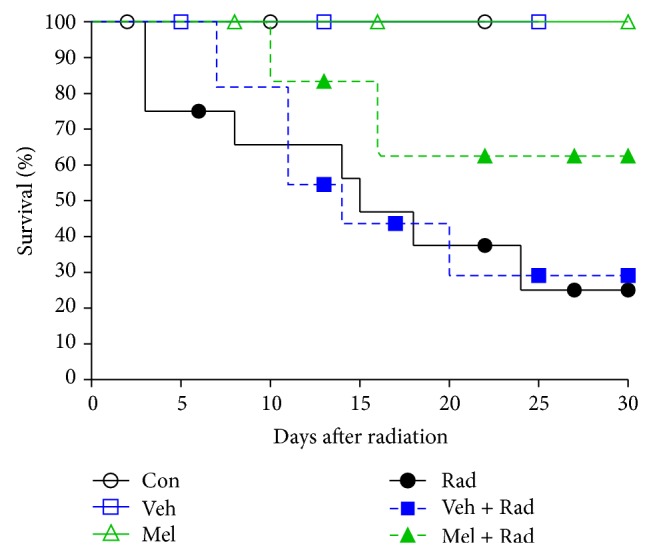
Survival of whole body irradiated rats pretreated with melatonin. Con: Control group, Veh: Vehicle only group, Mel: Melatonin only group, Rad: Radiation only group, Veh + Rad: Treated with vehicle and exposed to 10 Gy radiation, and Mel + Rad: Treated with 100 mg/kg melatonin before exposure to 10 Gy radiation.

Results from Figure 1 indicate that exposure of rats to lethal-whole-body radiation results in only 25% survival rate for Rad group and 29% survival rate for Veh + Rad group after 30 days. Treatment with melatonin in Mel + Rad group increased the survival rate up to 62%. Moreover, all rats in Con, Veh, and Mel groups survived after 30 days.

### 3.2. Body Weight

**Figure 2:**
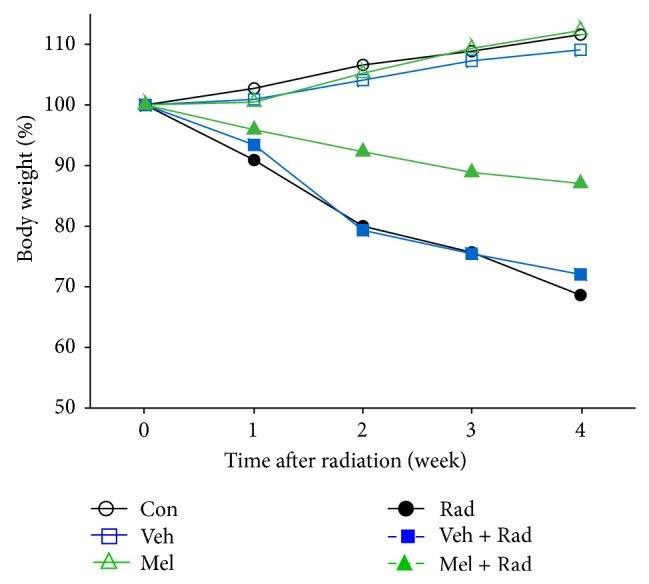
Percent change in body weight of rats. Con: Control group, Veh: Vehicle only group, Mel: Melatonin only group, Rad: Radiation only group, Veh + Rad: Treated with vehicle and exposed to 10 Gy radiation, and Mel + Rad: Treated with 100 mg/kg melatonin before exposure to 10 Gy radiation.

Figure 2 illustrates the maximum body weight loss for Rad group (31%) and then for Veh + Rad group (28%) whereas in Mel + Rad group it was minimum (13%) at the end of four weeks. Body weight for Con (11%), Veh (9%) and Mel (12%) groups increased after four weeks.

### 3.3. NO Levels

**Figure 3:**
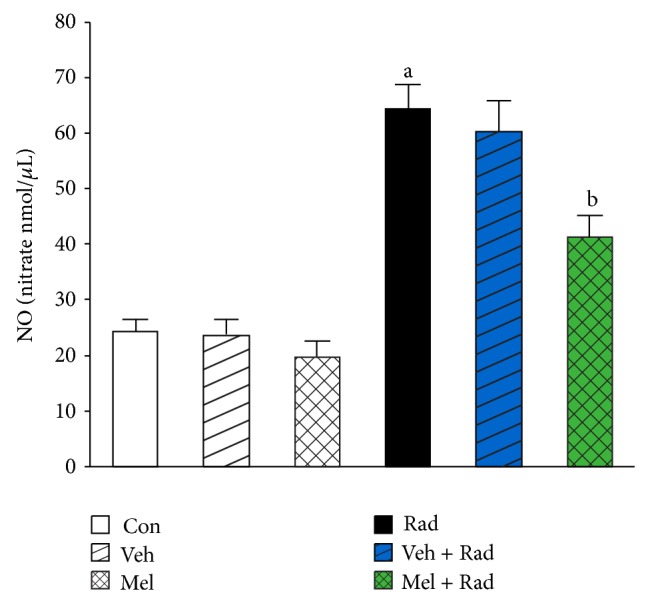
The effect of melatonin on NO levels in rats subjected to whole body gamma irradiation. Data represent mean ± standard error on the mean (SEM) for 5 animals per group. Con: Control group, Veh: Vehicle only group, Mel: Melatonin only group, Rad: Radiation only group, Veh + Rad: Treated with vehicle and exposed to 10 Gy radiation, and Mel + Rad: Treated with 100 mg/kg melatonin before exposure to 10 Gy radiation. ^a^ *P* < 0.001 when compared to Control group. ^b^ *P* < 0.001 when compared to the radiation only group.

As can be seen in Figure 3, irradiation significantly (*P* < 0.001) increases NO levels in Rad group (64.49 ± 4.51) compared to Control group (24.28 ± 2.35). Treatment with 100 mg/kg melatonin in Mel + Rad group (41.41 ± 3.94) significantly (*P* < 0.001) decreased NO levels in the serums of rats subjected to whole-body irradiation. There is no significant difference (*P* > 0.05) between NO levels in Control group versus Melatonin only (Mel) and Vehicle (Veh) groups. Moreover, data obtained from Rad and Veh + Rad groups was not statistically different (*P* > 0.05).

### 3.4. MDA Levels

**Figure 4:**
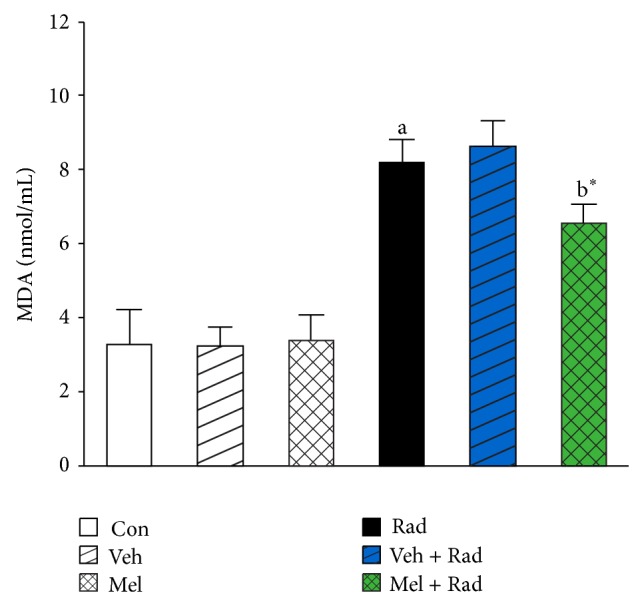
The effect of melatonin on MDA levels in rats subjected to whole body gamma irradiation. Data represent mean ± standard error on the mean (SEM) for 5 animals per group. Con: Control group, Veh: Vehicle only group, Mel: Melatonin only group, Rad: Radiation only group, Veh + Rad: Treated with vehicle and exposed to 10 Gy radiation, and Mel + Rad: Treated with 100 mg/kg melatonin before exposure to 10 Gy radiation. ^a^ *P* < 0.001 when compared to Control group. ^b^*^^ *P* < 0.01 when compared to the radiation only group.

As can be seen in Figure 4, irradiation significantly (*P* < 0.001) increases MDA levels in Rad group (8.19 ± 0.67) compared to Control group (3.29 ± 0.96). Treatment with 100 mg/kg melatonin in Mel + Rad group (6.58 ± 0.51) significantly (*P* < 0.01) decreased MDA levels in the serums of rats subjected to whole-body irradiation. There is no significant difference (*P* > 0.05) between MDA levels in Control group versus Mel and Veh groups. Moreover, data obtained from Rad and Veh + Rad groups was not statistically different (*P* > 0.05).

### 3.5. SOD Activity

**Figure 5:**
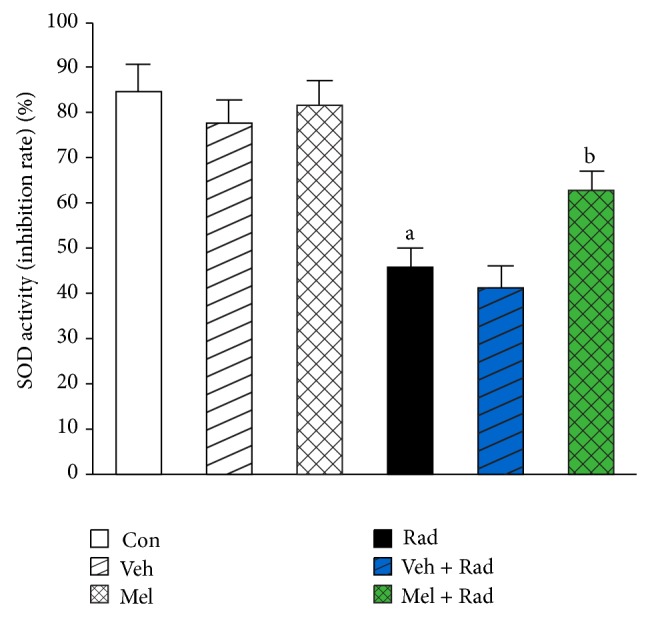
The effect of melatonin on SOD activity in rats subjected to whole body gamma irradiation. Data represent mean ± standard error on the mean (SEM) for 5 animals per group. Con: Control group, Veh: Vehicle only group, Mel: Melatonin only group, Rad: Radiation only group, Veh + Rad: Treated with vehicle and exposed to 10 Gy radiation, and Mel + Rad: Treated with 100 mg/kg melatonin before exposure to 10 Gy radiation. ^a^ *P* < 0.001 when compared to Control group. ^b^ *P* < 0.001 when compared to the radiation only group.

As shown in Figure 5, SOD activity in Rad group (45.91 ± 4.43) was significantly (*P* < 0.001) lower than Control group (84.71 ± 6.34). Treatment with 100 mg/kg melatonin in Mel + Rad group (62.96 ± 4.53) significantly (*P* < 0.001) increased the SOD activity in the serums of rats exposed to whole-body irradiation. SOD activity in Control group versus Mel and Veh groups was not significantly different (*P* > 0.05). Also, data obtained from Rad and Veh + Rad groups was not statistically different (*P* > 0.05).

### 3.6. GPx Activity

**Figure 6:**
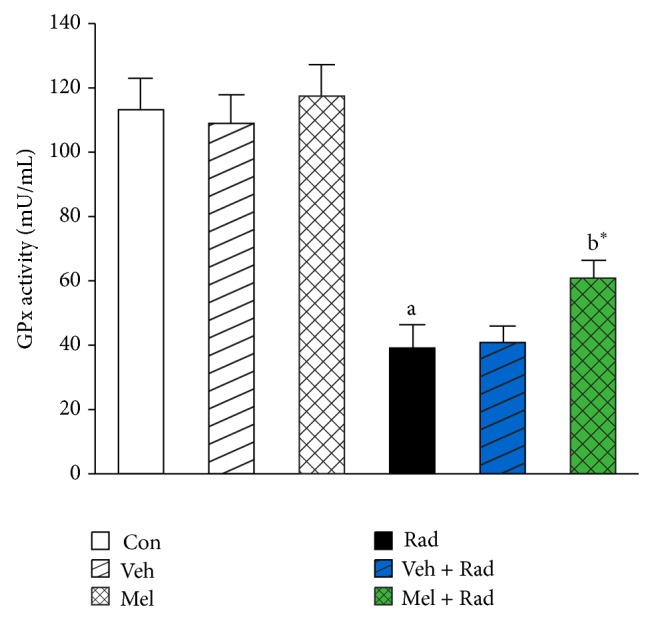
The effect of melatonin on GPx activity in rats subjected to whole body gamma irradiation. Data represent mean ± standard error on the mean (SEM) for 5 animals per group. Con: Control group, Veh: Vehicle only group, Mel: Melatonin only group, Rad: Radiation only group, Veh + Rad: Treated with vehicle and exposed to 10 Gy radiation, and Mel + Rad: Treated with 100 mg/kg melatonin before exposure to 10 Gy radiation. ^a^ *P* < 0.001 when compared to Control group. ^b^*^^ *P* < 0.01 when compared to the Radiation only group.

As shown in Figure 6, radiation significantly (*P* < 0.001) reduces GPx activity in Rad group (39.25 ± 7.39) when compared to Control group (113.30 ± 10.19). Treatment with 100 mg/kg melatonin in Mel + Rad group (60.75 ± 5.85) significantly (*P* < 0.01) improved the GPx activity in the serums of rats exposed to whole-body irradiation. GPx activity in Control group was not significantly (*P* > 0.05) different with Mel and Veh groups. The values of Rad and Veh + Rad groups were not also statistically different (*P* > 0.05).

### 3.7. CAT Activity

**Figure 7:**
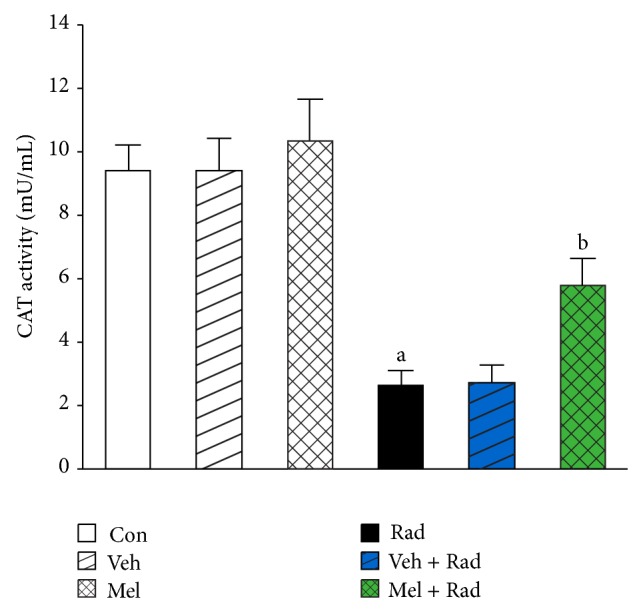
The effect of melatonin on CAT activity in rats subjected to whole body gamma irradiation. Data represent mean ± standard error on the mean (SEM) for 5 animals per group. Con: Control group, Veh: Vehicle only group, Mel: Melatonin only group, Rad: Radiation only group, Veh + Rad: Treated with vehicle and exposed to 10 Gy radiation, and Mel + Rad: Treated with 100 mg/kg melatonin before exposure to 10 Gy radiation. ^a^ *P* < 0.001 when compared to Control group. ^b^ *P* < 0.001 when compared to the Radiation only group.

As shown in Figure 7, CAT activity in Rad group (2.62 ± 0.51) was significantly (*P* < 0.001) reduced compared to Control group (9.42 ± 0.81). Treatment with 100 mg/kg melatonin in Mel + Rad group (5.80 ± 0.87) significantly (*P* < 0.001) increased the CAT activity in the serums of rats exposed to whole-body irradiation. CAT activity in Control group versus Mel and Veh groups was not significantly different (*P* > 0.05). Also, data obtained from Rad and Veh + Rad groups was not statistically different (*P* > 0.05).

### 3.8. TAC Levels

**Figure 8:**
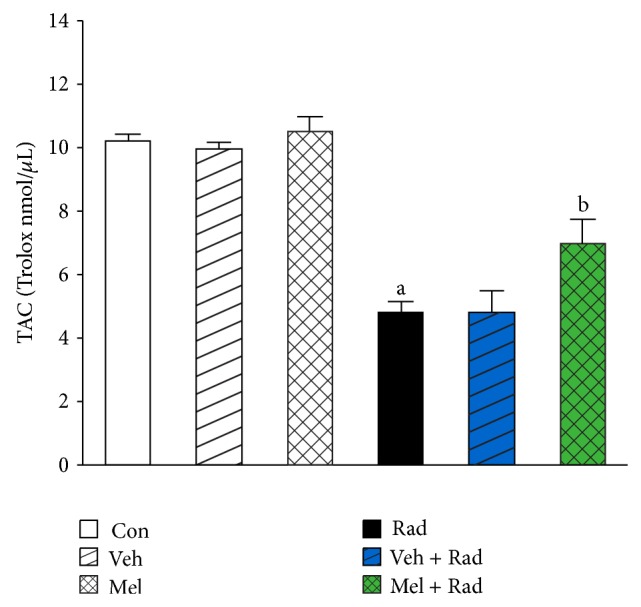
The effect of melatonin on TAC levels in rats subjected to whole body gamma irradiation. Data represent mean ± standard error on the mean (SEM) for 5 animals per group. Con: Control group, Veh: Vehicle only group, Mel: Melatonin only group, Rad: Radiation only group, Veh + Rad: Treated with vehicle and exposed to 10 Gy radiation, and Mel + Rad: Treated with 100 mg/kg melatonin before exposure to 10 Gy radiation. ^a^ *P* < 0.001 when compared to Control group. ^b^ *P* < 0.001 when compared to the Radiation only group.

As presented in Figure 8, TAC levels of Rad group (4.82 ± 0.35), were significantly (*P* < 0.001) depleted compared to Control group (10.21 ± 0.23). Treatment with 100 mg/kg melatonin in Mel + Rad group (6.97 ± 0.83) significantly (*P* < 0.001) elevated the levels of TAC in radiated group. TAC levels in Control group were not remarkably different (*P* > 0.05) with Melatonin only and Vehicle groups. Moreover, data obtained from Rad and Veh + Rad groups was not statistically different (*P* > 0.05).

### 3.9. Lymphocytes Count (LC)

**Figure 9:**
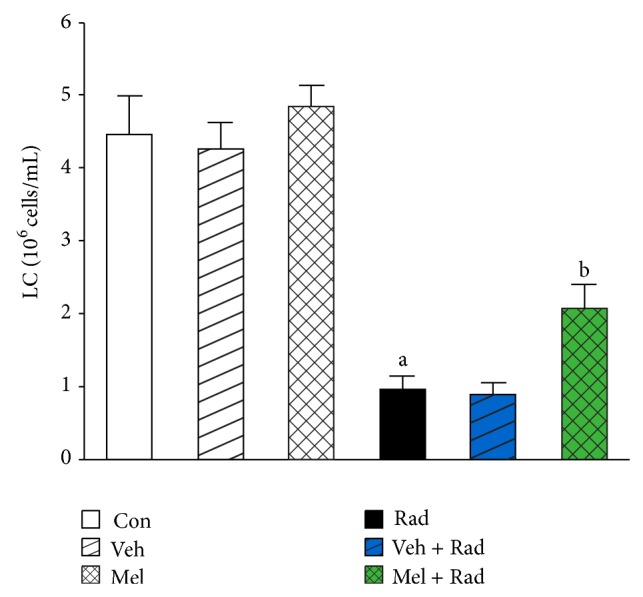
The effect of melatonin on LC in rats subjected to whole body gamma irradiation. Data represent mean ± standard error on the mean (SEM) for 5 animals per group. Con: Control group, Veh: Vehicle only group, Mel: Melatonin only group, Rad: Radiation only group, Veh + Rad: Treated with vehicle and exposed to 10 Gy radiation, and Mel + Rad: Treated with 100 mg/kg melatonin before exposure to 10 Gy radiation. ^a^ *P* < 0.001 when compared to Control group. ^b^ *P* < 0.001 when compared to the Radiation only group.

As presented in Figure 9, LC in Rad group (0.97 ± 0.20) was significantly (*P* < 0.001) lowered compared to Control group (4.47 ± 0.55). Treatment with melatonin significantly (*P* < 0.001) elevated the LC in Mel + Rad group (2.09 ± 0.33). LC in Control group was not remarkably different (*P* > 0.05) with Mel and Veh groups. The difference of Rad and Veh + Rad groups was not also statistically significant (*P* > 0.05).

### 3.10. Caspase-3 Activity

**Figure 10:**
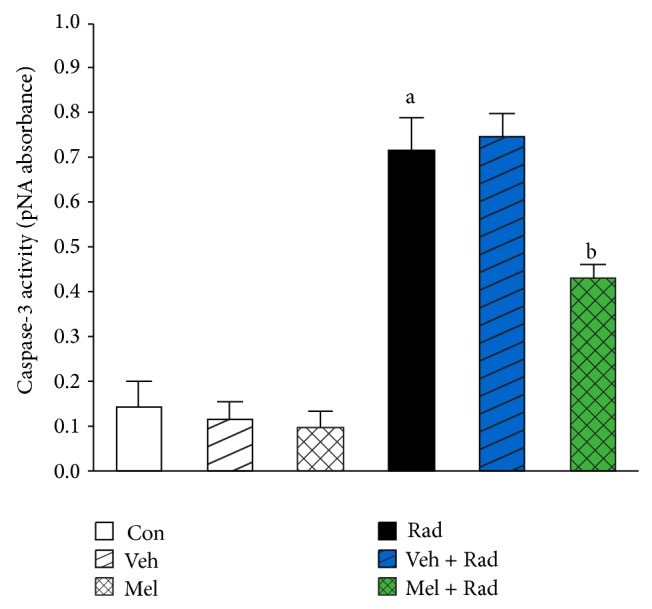
The effect of melatonin on caspase-3 activity in rats subjected to whole body gamma irradiation. Data represent mean ± standard error on the mean (SEM) for 5 animals per group. Con: Control group, Veh: Vehicle only group, Mel: Melatonin only group, Rad: Radiation only group, Veh + Rad: Treated with vehicle and exposed to 10 Gy radiation, and Mel + Rad: Treated with 100 mg/kg melatonin before exposure to 10 Gy radiation. ^a^ *P* < 0.001 when compared to Control group. ^b^ *P* < 0.001 when compared to the Radiation only group.

As presented in Figure 10, caspase-3 activity of Rad group (0.71 ± 0.08) was significantly (*P* < 0.001) increased compared to Control group (0.14 ± 0.06). Treatment with melatonin significantly (*P* < 0.001) decreased the activity of caspase-3 in Mel + Rad group (0.43 ± 0.03). Caspase-3 activity in Control group was not remarkably different (*P* > 0.05) with Melatonin only and Vehicle groups. Moreover, data obtained from Rad and Veh + Rad groups was not statistically different (*P* > 0.05).

## 4. Discussion

The blood tissue and lymphoid cells are vulnerable to injury induced by ROS and its damage may be life-threatening. Hence, drugs which protect the hematopoietic system from radiation-induced damage need to be identified [29]. Various chemical agents such as amifostine and other chemical compounds have been investigated as potential radioprotective drugs [9, 30]. However, the inherent toxicity of these compounds at the radioprotective doses warranted further search for safer and more effective radioprotectors [30–32].

Use of natural radioprotectors like melatonin has aroused increasing interest, since it may be beneficial in attenuating radiation damages in the following situations: diagnostic, therapeutic, and industrial settings; environmental background radiation present in soil, water, air, and so forth [14].

Mortality occurring after whole-body radiation, in addition to damages to hematopoietic and other tissues that have not been checked, may be attributed to inhibition of the immune system; that is, irradiation causes immunosuppression leading to death of the rats [23]. Endogenous infections also might have contributed to the death of the irradiated rats. The maximum mortality, in this study, occurred in Rad and Veh + Rad groups within thirty days after irradiation. Our results show that melatonin pretreatment provides protection against radiation-induced immune-suppression and mortality in rats.

In Rad and Veh + Rad groups of this study, significant decrease in body weight was observed after four weeks, which may be attributed to reduced food and water intake, loss of fluid and electrolytes through diarrhea, and diminished absorption capacity of the gastrointestinal (GI) tract [23]. The melatonin pretreated irradiated rats showed less change in body weight as compared to Rad and Veh + Rad groups.

In our present study, lethal-whole-body irradiation of rats to 10 Gy gamma radiation resulted in decrease in the TAC level as well as the antioxidant enzymes activity and increase in the NO and MDA levels of the serum. The increase in MDA levels may be due to the attack of free radicals on the fatty acid component of membrane lipids. In this study, we have observed a decrease in the activities of SOD, GPx, and CAT in Rad group. This decrease could be due to a feedback inhibition or oxidative inactivation of the enzyme protein caused by ROS generation, which in turn can impair the antioxidant defense mechanism, leading to an increased membrane LPO [33]. Thus, it seems that the activities of antioxidant enzymes are in close relationship with the induction of LPO, where the activities of SOD, GPx, and CAT declined with the increase in LPO [34].

Results obtained from our study indicated that treatment with melatonin (100 mg/kg) ameliorates the deleterious effects of 10 Gy irradiation by increasing the TAC level and antioxidant enzymes activity and decreasing NO and MDA levels. Therefore, melatonin suppressed the reduction of antioxidant enzymes activities (SOD, GPx, and CAT) and stimulated antioxidant defense system of rats by blocking the radiation-induced elevation on NO and MDA [16].

Our results showed 10 Gy irradiation induced damage to peripheral blood with decreases in LC. We studied the changes in LC in rats, since this is the important component of the immune system and affects the immune status of animals [35]. The LC in the Radiation only (Rad) group was significantly below Control and Melatonin only (Mel) groups. However, melatonin (100 mg/kg) by immune-stimulative action counteracted lethal-radiation-induced suppression of lymphocyte population.

As the caspase-3 activation is a common critical event for apoptosis [36], we studied the antiapoptotic effect of melatonin against lethal-radiation-induced apoptosis in lymphocyte cells. Caspase-3 appears to play its role after 10 Gy whole-body irradiation, leading to cellular apoptosis. Administration of melatonin prior to 10 Gy radiation inhibited caspase-3 activation.

On the other hand, data obtained in Melatonin only (Mel) and Vehicle (Veh) groups were close to Control group that showed insignificant differences (*P* > 0.05) in these groups. Thus, administration of vehicle and melatonin did not induce serious side effects and acute toxicity.

Researches in the last decade demonstrated that melatonin, by its free radical scavenging and antioxidative properties, ameliorates the radiation toxicity in different tissues [37]. Sharma et al. [38] showed that, due to its antioxidant properties, melatonin increased the immunity in squirrels, by protecting their hematopoietic system and lymphoid organs against 2.06 Gy X-ray-induced cellular toxicity. In their study, total leukocyte and lymphocyte counts (TLC and LC) in the peripheral blood and lipid peroxidation (LPO) status, superoxide dismutase (SOD) activities, total antioxidant status (TAS), and caspase-3 activity were measured in the spleens of squirrels. Pretreatment with melatonin prior to the irradiation significantly increased LC, TLC, SOD activity, and TAS status compared to irradiation exposed groups whereas LPO status and caspase-3 activity were decreased [38]. In another study, a radioprotective effect of melatonin against 5 Gy gamma irradiation during the reproductively active and inactive phases (RAP and RIP) of Indian palm squirrels was evaluated. Results showed that melatonin pretreatment significantly increased the LC and SOD activity and decreased the caspase-3 activity in the spleen of squirrels compared with irradiation group [2].

In our previous study [27], we investigated the possible radioprotective effects of 10 mg/kg melatonin against radiation- (2 and 8 Gy) induced oxidative damage on rats peripheral blood. Treatment with 10 mg/kg melatonin ameliorates harmful effects of 2 Gy irradiation by increasing antioxidant enzymes activity while it had not a significant protection against higher dose of 8 Gy [27]. The present study shows that 100 mg/kg melatonin administration ameliorated the immunity and the antioxidant status and lowered the NO and MDA levels in rats' peripheral blood exposed to 10 Gy gamma radiation. Therefore, it seems that radioprotective effects of melatonin are dose-dependent.

## 5. Conclusion

Results of the present study suggest that melatonin pretreatment provides protection against lethal-whole-body radiation-induced sickness, body weight loss, and mortality and it also ameliorates the immunity and the antioxidant status of rats. The hematopoietic cells can be protected from radiation-induced free radical damage by melatonin, which was evident in the increased number of lymphocyte in rats pretreated with melatonin.

On the other hand, melatonin treatment may have protected the lymphocytes from 10 Gy gamma radiation-induced apoptosis by inhibiting caspase-3 activity.
